# Crafting a public for geoengineering

**DOI:** 10.1177/0963662515600965

**Published:** 2015-08-27

**Authors:** Rob Bellamy, Javier Lezaun

**Affiliations:** University of Oxford, UK

**Keywords:** framing, geoengineering, performativity, public engagement

## Abstract

In a short period of time, climate ‘geoengineering’ has been added to the list of technoscientific issues subject to deliberative public engagement. Here, we analyse this rapid trajectory of publicization and explore the particular manner in which the possibility of intentionally altering the Earth’s climate system to curb global warming has been incorporated into the field of ‘public engagement with science’. We describe the initial framing of geoengineering as a singular object of debate and subsequent attempts to ‘unframe’ the issue by placing it within broader discursive fields. The tension implicit in these processes of structured debate – how to turn geoengineering into a workable object of deliberation without implying a commitment to its reality as a policy option – raises significant questions about the role of ‘public engagement with science’ scholars and methods in facilitating public debate on speculative technological futures.

## 1. Introduction

Over the last few years, ‘geoengineering’, or the possibility of intentionally manipulating the climate system to counteract global warming, has been added to the list of technoscientific issues subject to deliberative public engagement. Since 2009, a series of stage-managed forums have explored the prospects of carbon dioxide removal (CDR) and solar radiation management (SRM) – the two categories into which geoengineering proposals are commonly divided. Although most of these exercises have taken place in the United Kingdom, other forms of consultation have been tried elsewhere, and the number and range of public engagement activities are likely to expand in the future.

This article analyses this rapid and intense trajectory of publicization and explores the particular manner in which geoengineering has been incorporated into the field of ‘public engagement with science’ (PES). In a recent reflection on the future of PES research and practice, Stilgoe et al. (2014) have emphasized ‘the limits of evaluating individual exercises in their own terms’, calling instead for ‘critical, evaluative research that looks not at particular dialogues, but at the broader project of dialogic governance’ (p. 6). Here, we heed their advice and offer a second-order examination of how the dialogic governance of geoengineering has evolved in the United Kingdom since 2009. That year, the Royal Society published a landmark report, *Geoengineering the climate: science, governance and uncertainty*, and called for a broad and active programme of public deliberation on the desirability and conditions of use of climate engineering technologies. This recommendation was immediately taken up by the UK Research Councils, which sponsored a series of public forums where different geoengineering options were tackled in formal debate.

As Stilgoe et al. (2014) go on to argue, examining how projects of dialogic governance emerge around particular issues requires explicit attention to ‘the institutions that support public engagement as part of the experimental apparatus’ (p. 6), as well as, we might add, a reflexive examination on the part of PES scholars of the role that their own technical and methodological innovations play in the constitution of deliberative publics (Braun and Schultz, 2010; Felt and Fochler, 2010; Lezaun and Soneryd, 2007). We have therefore complemented our analysis with a number of interviews with PES scholars who were directly involved in the design, facilitation and analysis of public debates on geoengineering. Finally, we have drawn on our recent experience planning and managing deliberative workshops on geoengineering and climate change.^1^

What emerges from our analysis is what we will describe as a process of *unframing*. By this we mean a deliberate effort on the part of PES researchers to expand discursive and analytic frames of reference and thereby control the peculiar performative quality of public debate on geoengineering. An initial attempt to establish geoengineering as a discrete and well-characterized object of deliberation, most clearly expressed in the 2010 *Experiment Earth?* dialogue, was followed by a second wave of dialogic experiments that actively sought to problematize geoengineering as a self-contained ‘engagement matter’ (Irwin et al., 2013). This process of *unframing* was supported by a series of methodological innovations in the conduct of public deliberation – from a redefinition of the role of (natural–scientific) experts in articulating the matter under consideration, to a conscious effort to blur the boundaries of geoengineering as a distinct object of debate.

Similar examples of *unframing* are noticeable in the PES treatment of other controversial domains of technoscience, particularly as public forums move ‘upstream’ of research and development trajectories (Rogers-Hayden and Pidgeon, 2007; Wilsdon and Willis, 2004). What is remarkable about the case of geoengineering is the speed and deliberateness with which the PES community tried to reverse some of the discursive lock-ins implicit in early formulations of the issue as a public policy object. Our interviews with PES researchers suggest that their effort to place the issues within a broader, looser range of matters of concern was driven by the fear that geoengineering was being stabilized too quickly as a policy alternative, not least by the apparent success of PES initiatives in elucidating a set of stable public concerns and imaginaries. This fear was not exclusive to social scientists and PES researchers, however, as it was shared by many of the natural scientists involved in the initial scientific and technical appraisals of geoengineering proposals (Stilgoe, 2015). The result was a distinct methodological and political challenge: how to make planetary-scale climate engineering amenable for public deliberation without in the process making it ‘more real’ as a policy option.

## 2. From *Geoengineering the climate* to *Experiment Earth?*

The idea of curbing global warming by deliberately intervening into the Earth’s climate system has traditionally been at the margins of scientific and policy discussions.^2^ Over the last decade, however, it has lost some of its outlandish quality, as prominent voices within the scientific community have begun to demand a ‘plan B’ against climate change in the event that international efforts to reduce greenhouse gas emissions continue to falter. In 2006, the Nobel Prize–winning atmospheric chemist Paul Crutzen floated the idea of cooling the planet by injecting into the stratosphere sunlight-reflecting aerosols, presenting this form of geoengineering as a far-from-ideal but increasingly necessary response to the failure of policy makers to take effective action against climate change (Crutzen, 2006).^3^ In the aftermath of Crutzen’s intervention, the feasibility of different technologies for radical ‘climate remediation’ began to be openly discussed in a variety of scientific forums, a fact soon reflected in growing media interest in the subject (see Luokkanen et al., 2013; Porter and Hulme, 2013). It is in this context that the Royal Society decided to introduce a measure of scientific rigour in the rapidly expanding conversation on climate engineering.

The Royal Society 2009 report *Geoengineering the climate* was prepared by a working group that included social scientists and legal scholars. Presented in the lead-up to the climate negotiations in Copenhagen, the document achieved several feats in structuring the incipient debate on geoengineering.^4^ First, it carved out geoengineering as a specific and relatively self-contained object of public debate, offering what would quickly become a canonical definition: ‘the deliberate and large-scale intervention in the Earth’s climatic system with the aim of reducing global warming’ (The Royal Society, 2009: ix).

Second, it provided a taxonomy of geoengineering ‘methods’, organized around the distinction between CDR and SRM interventions. While CDR technologies seek to remove and store carbon dioxide from the atmosphere, SRM methods would operate by reflecting a proportion of sunlight away from the Earth. These two ‘classes’ of geoengineering were internally highly heterogeneous, however. Under CDR methods, the Royal Society considered, among others, the fertilization of the oceans to increase algal growth and CO_2_ uptake, the use of biomass energy coupled with carbon sequestration, the production of biochar and the direct capture of CO_2_ from ambient air. In turn, SRM options discussed in the report included the injection of sulphate aerosols into the lower stratosphere, the use of shields or reflectors in space and the enhancement of marine cloud reflectivity. The report went further than simply listing and classifying such hypothetical options and provided an initial evaluation of each on the basis of four criteria: affordability, effectiveness, safety and timeliness (The Royal Society, 2009: 6).

Third, the report placed the overall discussion of geoengineering within a set of considerations that emphasized the governance challenges posed by this response to climate change. Drawing an explicit parallel with other ‘emerging technologies’, the report drove home the message that ‘the acceptability of geoengineering will be determined as much by social, legal and political factors, as by scientific and technical factors’ (The Royal Society, 2009: 50). Finally, the report called for ‘an active and international programme of public and civil society dialogue … to identify and address concerns about potential environmental, social and economic impacts and unintended consequences’ (The Royal Society, 2009: xii), making research into public attitudes and meaningful public engagement a precondition for advancing a responsible R&D agenda.

To support the deliberations of the working group, the Royal Society commissioned a preliminary investigation into these public views. This research, conducted by the firm British Market Research Bureau, included four focus groups and a nationally representative telephone survey. While the survey method served to elicit rudimentary support or opposition to the forms of geoengineering under consideration, the focus groups method allowed a more nuanced discussion of attitudes towards climate change and climate politics before going on to discuss specific geoengineering ideas. Perceptions of geoengineering were shown to be generally negative, but complex and dependent upon the specific proposal under consideration (The Royal Society, 2009: 43). Importantly, the focus groups suggested that the prospect of geoengineering would not necessarily weaken the resolve to mitigate climate change through the reduction of greenhouse gas emissions and could instead galvanize those efforts – an observation that prompted the Royal Society to state that there was little empirical evidence to substantiate the ‘moral hazard’ argument against geoengineering (cf. Corner and Pidgeon, 2014; Gardiner, 2011).On the basis of this initial research, the report concluded that ‘further and more thorough investigations of public attitudes, concerns and uncertainties over geoengineering should be carried out in parallel with technological R&D, and accompanied by appropriate educational and knowledge exchange activities, to enable better informed debate and policy making’ (The Royal Society, 2009: 43).

In response to this call, the UK Natural Environment Research Council (NERC) joined forces with the Sciencewise Expert Resource Centre (the UK government–sponsored ‘national centre for public dialogue in policy making involving science and technology issues’), the Living with Environmental Change Partnership (a network of UK public-sector funders and users of environmental research) and the Royal Society to conduct the first large-scale deliberation exercise on geoengineering, the *Experiment Earth?* public dialogue. Designed and carried out by the research firm Ipsos MORI between February and May 2010, *Experiment Earth?* was a multi-pronged exercise designed to ‘identify and understand public views on geoengineering research and deployment, including its moral, ethical and societal implications’ (NERC, 2010: 1). Its largest component consisted of three reconvened deliberative workshops, which met for 2 full days of discussions over the course of 2 weeks. A section of those who participated in the workshops met for a final event at the National Oceanography Centre at the University of Southampton, where they had a chance to discuss their views with NERC staff, scientists and ‘climate stakeholders’. The dialogue also convened two ‘targeted discussion groups’, commissioned a qualitative online survey and sponsored three open events (one with school children, one a drop-in event at a science museum and one a discussion with a geoengineering researcher).

The dialogue activities in *Experiment Earth?* drew heavily on the taxonomical work of the Royal Society report. Facilitators divided the proposals into CDR and SRM varieties and presented participants with nine options to consider – five CDR (biochar, liming the ocean, iron fertilization, air capture and afforestation) and four SRM (stratospheric aerosol injection, mirrors in space, white roofs and cloud whitening) – offering for each a short list of potential risks and benefits. The discussions that ensued showed that participants had a low level of awareness or knowledge of geoengineering prior to their involvement in the exercise; yet at the same time, they were keen to discuss the pros and cons of specific proposals. Echoing the focus groups organized by the Royal Society, the discussions did not produce evidence of the feared ‘moral hazard’. The views expressed in the deliberative workshops indicated that ‘it was important to participants that geoengineering should not conflict with mitigation, and wherever possible should augment mitigation efforts’ (NERC, 2010: 2). Perceptions of individual technologies showed more support for CDR methods than for SRM alternatives, but again proved to be complex and dependent upon the specific proposal under consideration.

Sciencewise-ERC commissioned an official evaluation of *Experiment Earth?* Conducted by the environmental consultancy Collingwood Environmental Planning and using as its benchmark Sciencewise-ERC’s own principles for best practice in public dialogues on science and technology (context, scope, delivery, impact and governance), the evaluation concluded that the exercise had met the objectives stated by its sponsors. ‘In terms of scope’, the evaluation report noted,

‘whilst issues of climate change mitigation were raised by participants and there was some desire to situate the discussions in a wider context of environmental change, the participants felt able to raise key issues and felt the exchanges with scientists were extremely valuable’. (Orr et al., 2011: 1)

## 3. Setting the stage for a second wave of public engagement

After 2010, two UK Research Council initiatives provided the funding and institutional support for a further round of public deliberation on geoengineering. The Engineering and Physical Sciences Research Council (EPSRC) and NERC sponsored the Integrated Assessment of Geoengineering Proposals (IAGP), a programme designed ‘to conduct an objective, policy-relevant assessment of geoengineering proposals’. IAGP incorporated from the start a stream of deliberative workshops designed to ‘involve and engage with the lay publics and informed science-policy stakeholders’ (Corner et al., 2013). In parallel, the Stratospheric Particle Injection for Climate Engineering project (SPICE), an initiative funded by EPSRC, NERC and the Science & Technology Facilities Council, was launched to assess the feasibility of stratospheric aerosol injection. At the request of the Research Councils, SPICE developed a programme of public dialogue and stakeholder engagement around the intended deployment of a ‘testbed’ that would assess the feasibility of a stratospheric delivery system (Parkhill et al., 2013; Pidgeon et al., 2013; Stilgoe et al., 2013).

Two further independently funded public engagement exercises took place during the same period. First, a series of focus groups on SRM were carried out in Durham, Newcastle and London in December 2011 (Macnaghten and Szerszynski, 2013). Second, a group of researchers at the University of East Anglia affiliated with the IAGP project conducted in the summer of 2012 a hybrid analytic-deliberative appraisal of geoengineering proposals, consisting of two parallel strands of engagement: one for citizens and one for specialists (Bellamy et al., 2013, 2014).

These experiments in deliberative public engagement took place during a fairly short span of time (from the spring of 2011 to the summer of 2012; see Table 1) and were designed and carried out by a tightly knit community of UK-based PES researchers, with experts and facilitators often participating in, or advising on, more than one exercise. Significantly, this second wave was preceded by critical reviews of the *Experiment Earth?* public debate (Corner et al., 2011) and of geoengineering engagement and appraisal more generally (Bellamy et al., 2012). These reviews focused on the methodological and processual aspects of previous inquiries into public attitudes on geoengineering and identified problematic areas in the initial operationalization of geoengineering for deliberative dialogue.

**Table 1.** Deliberative public engagements with geoengineering.

Engagement	Methodology	Reference(s)
Royal Society focus groups (<September 2009)	Four focus groups of participants stratified by environmental beliefs and behaviours^a^	The Royal Society (2009)
Experiment Earth? Public dialogue (September 2009)	Three 2-day reconvened deliberative workshops (*n* ≅ 30 each) of socio-demographically representative participants (Birmingham, Cardiff, Cornwall); two ‘targeted discussion groups’ of young people and people at a risk of flooding (*n* = 10 each) (Birmingham, Cardiff); three open access events (Birmingham, Cardiff, Oxford)^a^	NERC (2010); see also Corner et al. (2011)
SPICE deliberative workshops (February 2011)	Three one- and a half-day reconvened deliberative workshops (*n* ≅ 10 each) of socio-demographically representative participants (Cardiff, Norwich, Nottingham)	Pidgeon et al. (2013) see also Parkhill et al. (2013), Stilgoe et al. (2013) and Stilgoe (2015)
SRM focus groups (December 2011)	Seven 3-hour focus groups (*n* ≅ 7 each) of socio-demographically representative participants (Durham, London, Newcastle) stratified by shared lifeworld experiences	Macnaghten and Szerszynski (2013)
IAGP deliberative workshops (Spring 2012)	Four 1-day deliberative workshops (*n* = 11 each) of socio-demographically representative participants (Birmingham, Cardiff, Glasgow, Norwich)	Corner et al. (2013)
Deliberative Mapping workshops (Summer 2012)	Two 2-day reconvened citizens’ panels (*n* ≅ 7 each) of socio-demographically representative participants (Norfolk) stratified by gender as part of a larger process also involving specialists	Bellamy et al. (2014); see also Bellamy et al. (2012), Bellamy et al. (2013) and Bellamy (2015)

IAGP: Integrated Assessment of Geoengineering Proposals; SPICE: Stratospheric Particle Injection for Climate Engineering; SRM: Solar Radiation Management.

a These engagements also included non-deliberative engagement elements, particularly surveys.

In Corner et al.’s (2011) evaluation of *Experiment Earth?* two discursive strategies of this exercise came up for particular criticism. First, facilitators had often used the notion of a climate emergency – an imminent ‘catastrophe’ or ‘crisis’ brought about by runaway climate change – to create decision-making scenarios for workshop participants. This framing, the review noted, not only avoided the thorny question of when (and by whom) such an ‘emergency’ might be declared, but also created a context that ‘artificially enhanced the acceptability of conducting research’ (Corner et al., 2011: 14) favouring in particular those geoengineering proposals that claimed to be fast-acting and highly impactful at reducing global temperature.^5^

Second, the use of the idiom of ‘naturalness’ to characterize certain geoengineering technologies was thought to have skewed public views on their acceptability (see also Corner and Pidgeon 2015). *Experiment Earth?* had concluded that participants preferred interventions they associated with the preservation of ‘natural systems’ or ‘natural processes’. Yet, the review argued, proposals were sometimes presented in a way that predetermined the public’s answer to this very question. Biochar, for instance, was repeatedly introduced as a ‘natural process’. Air capture and storage were likened to the use of ‘artificial trees’. The effect of stratospheric aerosol injections was explicitly compared to that of ‘volcanic eruptions’ (at a time when the Icelandic volcano Eyjafjallajökull had been a popular news item).

More generally, the review identified a tendency to structure the discussion around discrete and well-characterized technical options. *Experiment Earth?* had adopted the classification of geoengineering proposals presented in the Royal Society report, and much of the debate was organized around the risk–benefit parameters of individual technologies as defined in that report. According to the review, this had limited the ability of participants to explore geoengineering more broadly, whether in relation to alternative ways of tackling climate change or in terms of its broader political import. Before individual proposals were examined, the review argued, it was necessary to offer the public an opportunity to explore these larger issues at length.

In a similar vein, Bellamy et al.’s (2012) analysis of early geoengineering engagement and appraisal activities identified a rapid narrowing of framings. Geoengineering proposals had so far been considered in ‘contextual isolation’ of other options for tackling climate change. The deliberate, tacit or inadvertent exertion of power via framings had ultimately led to a premature ‘closing down’ of policy options (cf. Stirling, 2008), favouring those proposals that were presented as the most ‘technically effective’ – in particular stratospheric aerosol injection. Public participation in a high-stakes issue characterized by structural uncertainty, the analysis argued, demanded a mode of engagement more expansively framed than those of the surveys, interviews and experiments conducted so far. Geoengineering engagement and appraisal activities, the review concluded, should allow a greater diversity of discursive approaches so as to avoid premature sociotechnical lock-in and intractable conflict between divergent values and interests.

## 4. Deepening public engagement, *unframing* geoengineering

The second wave of public engagement exercises followed the advice of these two reviews and attempted to deactivate some of the assumptions and understandings built into the project of dialogic governance set in motion by the Royal Society report. The basic format of the new dialogues was similar to those conducted under *Experiment Earth?* – these were all invited ‘mini-publics’ carefully staged by expert facilitators – but the inputs provided to participants and the framings offered to position geoengineering as an object of discussion were very different from those of the previous inquiry. These modifications in the conditions of public deliberation amount, we will argue, to an effort to *unframe* geoengineering as a self-evident ‘engagement matter’.

A first element in the evolution towards a more open-ended constitution of the issue was the diminished role that scientific experts played in the second wave of deliberative workshops. *Experiment Earth?* had involved scientists and ‘STEM (Science, Technology, Engineering, and Mathematics) ambassadors’ in all of its events, and these experts had played a leading role in facilitating the discussion. In contrast, the public engagement events held in 2011 and 2012 sought to create spaces of deliberation that were largely free of expert involvement (i.e. other than social-scientific and facilitation expertise) or, in the case of the Deliberative Mapping exercise, challenging the power relations between experts and lay participants by including both but treating them symmetrically (Bellamy et al., 2014).

In the workshops conducted in the context of the SPICE project, for instance, experts were invited to participate in the initial discussions but were excluded from the second-day debates. Similarly, the facilitation of the focus groups on SRM conducted in Durham, Newcastle and London did not include any natural scientists, a decision made ‘to ensure the discussion was not framed by experts’ (Macnaghten and Szerszynski, 2013). Even when experts were on hand to provide technical answers, their responses were carefully managed to make sure they served to elicit further (lay) views, rather than to provide definitions that would effectively close particular lines of inquiry. A member of the team that conducted the deliberative workshops for the SPICE project describes as follows the role of the scientific expert who participated in those discussions:

We quite carefully managed her, so before she was allowed to answer anything and the natural thing happened that an expert steps in and everybody starts deferring to her, so we used to just stop her dead before she could answer any questions. The facilitators would prompt and probe with the publics as to why they were asking what they were asking, what they really wanted to know, really thoroughly exploring with them what was the basis of their questions and once we had done that then we would say: ‘Right Julia [pseudonym], now please go ahead and answer’. So that was quite carefully managed and in the end I got her so well trained that I wouldn’t have to say stop; she would just stop herself and go: ‘So tell me why you’re interested?’ (Interview 2)

The expert in question describes the adoption of this restrained role as her personal ‘learning curve’:

For me, personally, the only technical difficulty for me, the only learning curve, was how to communicate with people. At the end of the day I am still a physical scientist. I do like it to be right … so learning to let go of that. I had to also learn how to facilitate, learn how to sit on your hands when someone’s saying carbon monoxide when they mean carbon dioxide, because what’s important is not that they said the wrong thing, it’s the point that they’re making, and if you do correct them then you shut down their engagement. (Interview 3)

While the scientists involved in *Experiment Earth?* had (in the words of the official report) ‘revealed (consciously or unconsciously) their own opinions about the technologies under discussion’ (NERC, 2010: 78), the careful (self)restraining of expert input in this second wave of deliberation exercises shifted the balance away from the clarification of technical issues and towards a deeper articulation of the concerns of lay participants. As the expert involved in the SPICE workshops puts it,

We tried to not answer technical stuff, not play ‘poke the monkey in the cage’ kind of thing with the expert. Which is: you’re trapped in a room with someone who’s apparently an expert on climate change so I’m going to ask them all those questions I wanted to ask, like how efficient is a wind farm and is there any point in me unplugging my mobile phone from the charger at night? We tried to avoid that as much as possible and they would often be interrupted with, once they’d posed their question, ‘Can I just ask you why you’re interested in that?’ because that’s what the facilitators were interested in: ‘Why is it important to you?’ (Interview 3)

**Figure 1.**
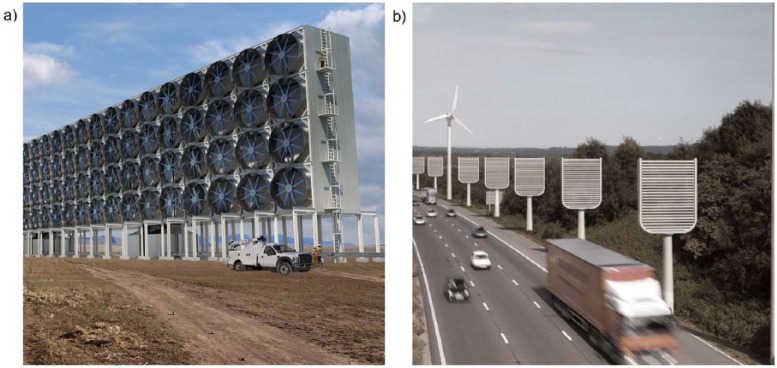
Artists’ impressions of air capture and storage devices.^6^ (a) ‘Giant fans’. Credit: Carbon Engineering Ltd. (b) ‘Artificial trees’. Courtesy Institute of Mechanical Engineers.

In parallel to this re-positioning of scientific expertise, there was a conscious effort to downgrade the status of different geoengineering options. What in *Experiment Earth?* had been presented as geoengineering ‘techniques’ or ‘technologies’ became ‘proposals’ in the Deliberative Mapping project or ‘ideas’ in the SPICE workshops. A striking example of this attempt to make geoengineering seem less presently real, while holding it sufficiently in place to eliciting a clear set of public reactions to it, is the use of visual materials in the discussions. *Experiment Earth?* had made extensive use of images to depict what different geoengineering alternatives might look like in the future. The facilitators in the new engagement exercises were careful to identify these images as ‘artist’s impressions’ of what were essentially fictional technologies. One of the facilitators in the SPICE workshop described their use of visual imagery in those discussions as follows:

We used the ones that were already available, but what we did take great pains to do is to make sure that we labelled everything as such, and said that these are not actual photographs, because some of them do look kind of like photographs, and there were certain ones that we wouldn’t use … I mean, for example, I guess, there are some CDR sort of artist impressions that do make them look like trees, so we didn’t use those sorts of things. We used the sort of ones that look like giant fans really (Figure 1). Well, certainly that is the way that a lot of people do envision they will look like, rather than more like the things next to the motorways, where they look kind of like graters and look quite in keeping with the landscape. (Interview 2)

This careful positioning of images was meant to qualify the ‘reality effect’ that any visual representation of geoengineering was bound to have. The images of CDR technologies presented to participants were all equally fictional; the choice of image thus reflected a preference for a certain aesthetic register to represent the future of geoengineering – or, more accurately, of what a future with geoengineering in it might look like. The ‘grater’ conveys a future in which geoengineering is a design-conscious intervention, ‘in keeping’ with a landscape that appears futuristic in a suitably contemporary way (the artefacts appear relatively small next to the giant wind turbines, and their sleek curvature resembles that of a portable communication device). The ‘giant fan’, in contrast, appears bulky and intrusive. The choice of this image to depict what CDR might look like was thus a choice for a more obtrusive, less streamlined future, one in which geoengineering stands out more starkly from the environment, reminding us perhaps of our industrial-era environmental sins.

Finally, and most importantly, the engagement exercises in this second wave all made a clear effort to situate ‘geoengineering’ within broader discursive fields. All the PES researchers we interviewed cited this as their foremost preoccupation in designing a new round of public discussions. As a member of the Deliberative Mapping project team puts it,

One of the big things for me coming into the geoengineering space is around the framing of these kinds of processes and how at the time all public engagements on geoengineering were focussed around particular geoengineering technologies or comparing particular geoengineering technologies within themselves. So the big thing probably that was brought into this space was to take this kind of opening up approach, to open up the framing and say ‘Well, actually what is geoengineering trying to attend to? What kind of problems is it trying to address?’ (Interview 4)

This ‘opening up’ took different forms in each of the deliberative exercises, but the goal was always to break out of the set of assumptions so forcefully inscribed in the format of public dialogue that emerged out of the Royal Society report. The workshops conducted by IAGP, for instance, introduced geoengineering as part of a ‘societal responses to climate change’ frame, rather than treating it as a singular object of debate (Corner et al., 2013). The Deliberative Mapping project forced participants to consider geoengineering proposals symmetrically alongside mitigation options and adaptation (Bellamy et al., 2014). The SRM focus groups went even further, tentatively de-coupling geoengineering from climate change altogether, pursuing instead the topic within a broader consideration of ‘climate technics’ that focused on their compatibility with different social worlds and political systems (Macnaghten and Szerszynski, 2013).

Several facilitation techniques were used to expand the frame of reference of these discussions. Geoengineering was never introduced at the start of the discussion, with participants having undergone topic-blind recruitment in all cases. In the IAGP workshops, geoengineering was introduced alongside mitigation and adaptation after a presentation on climate change (Corner et al., 2013). In the SRM focus groups, geoengineering was first introduced 70 minutes into the debate, after a long discussion on participants’ experiences of weather and climate (Macnaghten and Szerszynski, 2013). The Deliberative Mapping project went further still, allowing participants themselves to frame initial deliberations on global environmental challenges, until climate change and the range of responses to it, including some ideas that could plausibly be construed as geoengineering, emerged ‘naturally’ during the course of the discussions (Bellamy et al., 2014). In the words of one of our interviewees, these facilitation tactics ‘resituated geoengineering in the context of other climate policy options’. This, according to the same interviewee, was in deliberate contrast to the manner in which the Royal Society had positioned the issue in its 2009 report:

The Royal Society report had snatched [geoengineering] away from the climate debate, and the Royal Society has particular issues with talking about climate, and talked about [geoengineering] as its own technology to be assessed. (Interview 1)

In contrast, this new round of deliberative experiments implemented deliberative designs and facilitation tactics that constrained the discursive availability of ‘geoengineering’ as a self-standing matter – a technology, or bundle of technologies, that could be evaluated on its own merits – seeking to reconstruct the link between the promises of control and remediation implicit in climate engineering imaginaries and the larger question of mankind’s relation to climate and climate change. As we will see below, this process of *unframing* was a deliberate response to the perceived role that public engagement was beginning to play in reifying geoengineering as a tangible technological future.

## 5. Public engagement and the reality of geoengineering

PES researchers have long recognized and criticized the perception among policy makers that officially sponsored deliberation might offer a shortcut to the public acceptance of new or controversial technologies (Wynne, 2006). In the case of geoengineering, there was a risk that advocates of a ‘plan B’ for how to tackle climate change would see a round of public engagement exercises as a pro forma step before launching a full-blown programme of R&D activities. In the words of one of our interviewees,

This wasn’t just an academic problem; it was a practical problem because the way that the social sciences get used within that space could have implications for how people think of questions around public acceptability and the concern that I had then was that I felt there was a danger that the kind of public engagement element could be seen purely in process terms: So, okay, we need to do some public engagement. We’ve done the public engagement, so now we can get on and do the experiment without really seriously thinking about well, what’s the substantive nature of the concerns associated with that technology and what might that mean in terms of whether this test should go ahead or not? (Interview 6)

Geoengineering brought a particular twist to this problem. In 2011 and 2012 the UK government was still undecided about whether geoengineering should be the recipient of public research funds. The Royal Society report had recommended a 10-year programme of research ‘on geoengineering and associated climate science’ to the tune of £10 million per annum, and the social-scientific work sponsored by the Research Councils was meant to clarify the challenges to be faced by a full-blown commitment to the development of climate engineering technologies. In this context, public deliberation could play an outsize role, most significantly by ‘normalizing’ geoengineering as both a matter of public debate and a suitable beneficiary of state support. This gave social scientists in general, and PES researchers in particular, an unusual amount of influence, but that influence came with an additional level of anxiety. In the words of a researcher who was closely involved in several of these initiatives,

The speed with which geoengineering has gone from being entirely speculative through the Royal Society report and the various research projects subsequently into something that is seen as very real – even though there is actually no additional knowledge on which to base that reification – is terrifying. (Interview 1)

As this statement suggests, public dialogue could play here not merely a legitimizing role, but a performative one as well. That is, it could make geoengineering appear a real and viable course of action, even in the absence of systems to properly address the scientific challenges and governance dilemmas posed by the very idea of planetary ‘climate control’. This gave public engagement an additional political dimension. In the words of the same interviewee,

The thing I think we all need to be acutely aware of is that in thinking about and doing public engagement, even if we are conscious of the risk of manipulating a particular consensual public, we are also making the technology seem more likely. (Interview 1)

To those committed to public dialogue on geoengineering, this posed a pressing challenge: how to create a space for discursive engagement with the issue – how to make the subject tractable to discussion – without in the process enhancing its consistency as a policy object. This conundrum is to some extent true of any public dialogue that moves ‘upstream’ in the R&D process, but it operated with a vengeance here. Public engagement exercises were taking place at a time when geoengineering seemed to be quickly becoming part of mainstream scientific and policy discussions. The Royal Society report was a turning point in that regard, and it was soon accompanied by several initiatives on both sides of the Atlantic that reinforced the trend. The inquiry into geoengineering sponsored jointly in 2009 by the Science and Technology committees of the US House of Representatives and the UK House of Commons, the 2010 Asilomar International Conference on Climate Intervention Technologies and the inclusion of geoengineering in the Fifth Assessment Report by the Intergovernmental Panel on Climate Change were other markers of the rapid ‘normalization’ of geoengineering in policy circles. It is in this context that PES activities took a distinctive turn, away from the elucidation of public views on individual geoengineering proposals (or on geoengineering as a whole), and towards deliberative processes that consciously challenged the boundaries of geoengineering as a discrete topic of engagement.

We have referred to this turn as a process of *unframing*. By this we mean not only an effort to resist and reverse early discursive lock-ins in the definition and appraisal of geoengineering, but a more ambitious struggle to problematize its stabilization as a self-contained matter of concern and policy object. Any attempt to position geoengineering (or any other subject) for public discussion involves acts of framing and re-framing since it unavoidably establishes a set of assumptions that restrict the scope of debate and regulate the sequence of arguments and counter-arguments (cf. Entman, 1993). The specificity of *unframing* comes, in our view, from the very particular relationship that exists between the articulation of geoengineering for public discussion and its growing consistency as a policy alternative. The urgency of the threat posed by climate change, and the well-known obstacles facing effective global action to reduce greenhouse gas emissions, create enormous pressure to at the very least define the conditions under which research on a climate engineering alternative could proceed. By probing the boundary between development and deployment, and by considering the complex interaction between the pursuit of geoengineering and the determination to make the sacrifices required to tackle the causes of global warming, the second wave of deliberative exercises complicated any direct or unambiguous translation of ‘public views’ as an endorsement for any singular course of action.

This is not to suggest that the opinions elicited in these deliberative forums were any less ‘actionable’ or policy-relevant than those obtained in previous public consultation exercises. But these views did not amount to a set of preferences (or misgivings) vis-à-vis an already prefigured choice. Rather, they tended to express more complex constellations of environmental and political concerns, within which geoengineering might feature as either problem or solution (or both). These public deliberations thus served to call attention to the conditions that had prompted interest in geoengineering in the first place: the intensification of anthropogenic climate change, and the technological and political factors that limit our ability to control its causes. They sought to relate this technological future back to the current failures and predicaments that give it its promissory quality, hoping to transform a highly speculative proposition into an opportunity to confront the challenges of the present.

## 6. Discussion

Geoengineering constitutes a very particular object in the historical trajectory of the field of ‘public engagement with science’. In some respects, it represents a high-water mark in terms of the influence of structured mini-publics on policy-making, at least in relation to the formulation of a governmental research funding agenda in the United Kingdom. The 2009 Royal Society report and the subsequent forums sponsored by the UK Research Councils were attempts to map societal concerns and expectations in advance of any substantial public (or private) investment in the development of purposefully designed climate engineering technologies. Geoengineering can thus plausibly be seen as an area where the demand for ‘upstream’ public engagement has been met, if we understand by this the articulation of a space of participation that precedes the development of the science and technology in question.^7^

At the same time, the case of geoengineering has made visible some of the challenges and paradoxes implicit in this vision of ‘early stage’ deliberation. Because the project of dialogic governance inaugurated by the Royal Society report unfolded largely in the absence of a spirited public debate on these issues, designers and facilitators of public engagement events had to construct the topic of geoengineering from scratch, so to speak, for participants who were largely ignorant of the subject and had to be equipped in the course of the deliberation to discuss the matter at hand (Corner et al., 2012). The relatively small community of ‘dialogue specialists’ and PES researchers involved in these exercises was thus afforded considerable leeway in the framing of the topic.

The familiar challenge of how to articulate a controversial technology for public discussion was here exacerbated by the particular connotations of geoengineering as a mode of intervention. If, as Stilgoe (2015) encourages us to do, we resist reading ‘geoengineering’ as a noun – the descriptor of an eclectic inventory of technologies – and treat it as a verb, a term that designates an ongoing, world-shaping project oriented towards the manipulability of the climate, we begin to appreciate the added burden on PES practice in this area. Geoengineering, including *talk* about geoengineering, is an activity that radically respecifies our relationship to the Earth’s climate by introducing in our calculations the possibility (and expectation) of technological control (see also Hulme, 2014).^8^

Seen in this light, geoengineering research, and research on geoengineering (including on the evolving set of ‘public attitudes’ that might hinder or accelerate its progress), becomes expressly performative. While this performative dimension of public engagement has been noted in relation to other emerging technologies (see, for instance, Delgado et al., 2011; Irwin, 2006; Myers, 2004), the situation is compounded here by the fact that the possibility under discussion is always being contrasted implicitly or explicitly with an alternative, conventional mitigation via reduction of greenhouse gas emissions, that is universally understood to present enormous technical and political challenges. Against this background, geoengineering emerges de facto as a technical fix; it acquires the character of an insurance against uncontrolled climate change – an insurance that appears all the more realistic by virtue of being elaborated in a purely discursive manner and within the relatively safe confines of public engagement forums.^9^

For all these reasons, the engagement exercises in the second wave of deliberations discussed above were forced to spend much time and effort re-positioning geoengineering as an object of public debate. They did so by keeping the issue firmly within a broad constellation of approaches to climate change that included, crucially, the current and future prospects of mitigation endeavours. Furthermore, in these exercises, we observe a shift in the role of (natural) scientific expertise in structuring public dialogue and a greater effort to manage the ‘reality effect’ of the inputs used in the discussions. The result was always a balancing act: geoengineering was to be turned into an explicit matter of debate, without in the process giving it too much credence as a realistic, or even real, policy option.

One could argue that by addressing explicitly the performative aspects of its own practice, PES scholarship continues to evolve beyond schemes that treat the assembling of deliberative publics as external to processes of technoscientific development. The result of this evolution is a more finely balanced position vis-à-vis science policy and the materialization of technological futures. Elucidating public views on a controversial technology is always part and parcel of that controversy. PES reveals its true potential, and its peculiar predicament, when it sees itself as gathering temporary collectives around an uncertain and volatile issue, a mode of intervention that is expected to stabilize both the object *and* the subject of engagement.

This is a situation where traditional criteria for evaluating the success of PES interventions, such as the representativeness of the deliberative mini-public or the disinterestedness with which the issues are articulated for debate, lose some of their relevance, as it becomes clear that the very constitution of a public able to reflect on the matter in question carries with it a certain commitment to the reality of the technology under consideration. This challenge of performativity was particularly salient in the case of geoengineering since the absence of alternative venues for the emergence of publics and counter-publics transformed stage-managed exercises in deliberation into key sites for the realization of this speculative technological future.

Our research indicates that PES researchers are highly reflexive practitioners of this art of public-making. This reflexivity, the care with which the reality of geoengineering was qualified in public engagement exercises at a time when this mode of intervention seemed to be emerging as a appealing response to climate change, suggests that a full reckoning with performativity might be the wellspring of new and possibly more nuanced critical capacities for the field (cf. Callon et al., 2011; Irwin et al., 2013; Michael, 2012). A curatorial attention to the publics we help bring into being comes with a new understanding of their desired qualities and imposes a new set of demands and expectations on the facilitators of public dialogue. It introduces new criteria of procedural validity and political responsibility – a new deliberateness, so to speak, in the design and conduct of deliberation processes. This deliberateness is particularly appropriate when, as in the case discussed here, the publics in question are assembled to reflect on the means and ends of new forms of planetary care.

## Notes

1. We read and reviewed the official reports, evaluations and scientific publications that have emerged from the public deliberations on geoengineering conducted in the United Kingdom between 2009 and 2012 (see Table 1 for a complete list). Additionally, we interviewed six ‘public engagement with science’ (PES) scholars who were directly involved in the design, conduct and interpretation of these exercises. The interviews were conducted between February and April 2014. We circulated an early draft of this article among our interviewees and to an extended group of PES researchers with experience in the geoengineering debate. We have incorporated some of their responses in the final version of the article. One of the authors of this article (R.B.) led one of the six engagement exercises discussed in the article: the Deliberative Mapping workshops conducted in Norfolk in the summer of 2012. In parallel to writing this article, the authors designed and conducted a further set of workshops on geoengineering governance imaginaries. This latter work was carried out in the context of the Climate Geoengineering Governance project, funded by the UK Economic and Social Research Council (ESRC) and the Arts and Humanities Research Council (AHRC).

2. For an account that places the current interest in geoengineering within a longer history of weather and climate modification, see Fleming (2010). Locher and Fressoz (2012) offer an even longer genealogy of the idea that ‘a certain pliability’ might indeed characterize the climate – a form of ‘environmental reflexivity’ that opened the door to multiple modalities of intervention in the modern era.

3. In a sort of afterthought, Crutzen (2006) noted the role that public opinion would have to play in making stratospheric sulphur injections a politically acceptable proposition: ‘Building trust between scientists and the general public’, he pointed out, ‘would be needed to make such a large-scale climate modification acceptable, even if it would be judged to be advantageous’ (p. 217).

4. For a ‘behind-the-scenes’ look at the preparations of the report, see Stilgoe (2015: Chapter 4).

5. On the use of emergency framings in the geoengineering debate, see Hulme (2014), Amelung and Funke (2015), Nerlich and Jaspal (2012) and Markusson et al. (2014).

6. The images actually represent two technically distinct variants of air capture, namely, (a) alkaline sorbent scrubbing and (b) moisture-swing sorption.

7. In addition to the role played by PES activities, the social sciences have been a key contributor to early scientific discussions on geoengineering. According to Linnér and Wibeck (2015), by 2013 social-scientific publications on geoengineering had begun to outnumber contributions from the natural sciences.

8. As Szerszynski has argued, this implies an understanding, or ‘enframing’ as he calls it, of climate change as the object of a broad set of ‘climate technics’ – a project that has, in his view, an obvious soteriological subtext (Szerszynski, 2010). On the deep continuity of geoengineering with other forms of climate science, see Stilgoe (2015: 64–66).

9. Alfred Norman has noted how ‘speculative ethics’ allows imagined futures to ‘overwhelm the present’ and thereby creates a mandate for technological adoption by projecting a world that appears free of the alternative courses of action evident in the contemporary situation (Nordmann, 2007). Speculative public engagement can be said to operate in a similar fashion, defining as its object a novel, spectacular technology, and abstracting it from present-time opportunities and constraints that would otherwise qualify its appeal.
